# Association of ambient temperature with the outcomes in witnessed out-of-hospital cardiac arrest patients: a population-based observational study

**DOI:** 10.1038/s41598-019-50074-7

**Published:** 2019-09-16

**Authors:** Chiwon Ahn, Jihoon Kim, Wonhee Kim, In Young Kim, Hyun Young Choi, Jae Guk Kim, Bongyoung Kim, Shinje Moon, Hyungoo Shin, Juncheol Lee

**Affiliations:** 1 Department of Emergency Medicine, Armed Forces Yangju Hospital, Yangju, Republic of Korea; 2 Department of Biomedical Engineering, College of Medicine, Hanyang University, Seoul, Republic of Korea; 3 Department of Thoracic and Cardiovascular Surgery, College of Medicine, Hallym University, Chuncheon, Republic of Korea; 4 Department of Emergency Medicine, College of Medicine, Hallym University, Chuncheon, Republic of Korea; 5 Department of Internal Medicine, College of Medicine, Hanyang University, Seoul, Republic of Korea; 6 Department of Internal Medicine, College of Medicine, Hallym University, Chuncheon, Republic of Korea; 7 Department of Emergency Medicine, Hanyang University Guri Hospital, Guri, Republic of Korea; 8 Department of Emergency Medicine, Armed Forces Capital Hospital, Seongnam, Republic of Korea

**Keywords:** Cardiovascular diseases, Cardiovascular diseases

## Abstract

This study aimed to identify the association between ambient temperature (AT) and patient outcome of witnessed out-of-hospital cardiac arrest (OHCA) occurring outdoors. This retrospective nationwide, population-based cohort study recruited witnessed adult OHCA patients in South Korea from January 2012 to December 2016. Meteorological data of 17 metropolitan cities and provinces were retrieved from the Korea Meteorological Administration database. Primary outcome was sustained return of spontaneous circulation (ROSC) in hospital. Secondary outcome was survival to hospital discharge. By the standard of quartile categories of AT (Q1 = 7.1 °C; Q2 = 17.7 °C; Q3 = 23.5 °C), three comparative analyses for ROSC and survival were performed between low and high AT groups. Propensity score matching (1:1) was performed for both AT groups. Among the 142,906 OHCA patients, 1,295 were included. In the multivariate analysis for matched groups by the standard of 7.1 °C (Q1), proportion of ROSC was significantly higher in the high AT-Q1 group than in the low AT-Q1 group (adjusted odds ratio [aOR] 2.02, 95% confidence interval [CI] 1.19–3.44). No significant difference in survival was shown between both AT-Q1 groups (aOR 1.24, 95% CI 0.61–2.52). In the standard of 17.7 °C (Q2) and 23.5 °C (Q3), no significant differences in ROSC and survival were found between the low and high AT groups. In conclusion, no obvious correlation existed between AT and patient outcomes such as sustained ROSC or survival to discharge in this study.

## Introduction

Out-of-hospital cardiac arrest (OHCA) is a notable public health problem, with almost 400,000 Americans experiencing OHCA annually^1,2^. The significant issue is increasing survival through proper treatment and management^1^. In-hospital cardiac arrest (IHCA) has a better outcome due to shorter arrest with rapidly treatable cause^3,4^, whereas OHCA typically occurs in patients without witnesses, in which bystander cardiopulmonary resuscitation (CPR) could not be performed, which could lead to a lower survival rate^1,5^. Additionally, shockable cardiac rhythm and spontaneous circulation in the field have a significant influence on survival in OHCA^5–7^. These factors are well known to affect the outcome of patients with OHCA^1,5,7^.

Several recent studies have suggested that cold and hot weather is related to the occurrence of OHCA^8–11^. However, the relationship between ambient temperature (AT) and OHCA prognosis is unclear. Fukuda *et al*. showed that seasonal AT affected the outcome of OHCA patients^8^. However, it is not clear whether AT directly affected the status of OHCA patients, because they could be affected by the temperature around the place of arrest occurrence than by the AT. Thus, it is necessary to limit the study population to investigate the effects of AT on OHCA.

Records of all of the OHCA patients who are transported to the hospital via emergency medical service (EMS) are recorded in the database. In addition, meteorological data as public information were easily accessible from the Korea Meteorological Administration database. To investigate the association prognosis of OHCA with AT, we performed the study on patients with cardiac arrest occurring outdoors with combination of weather data.

## Results

### Patient characteristics

**Figure 1:**
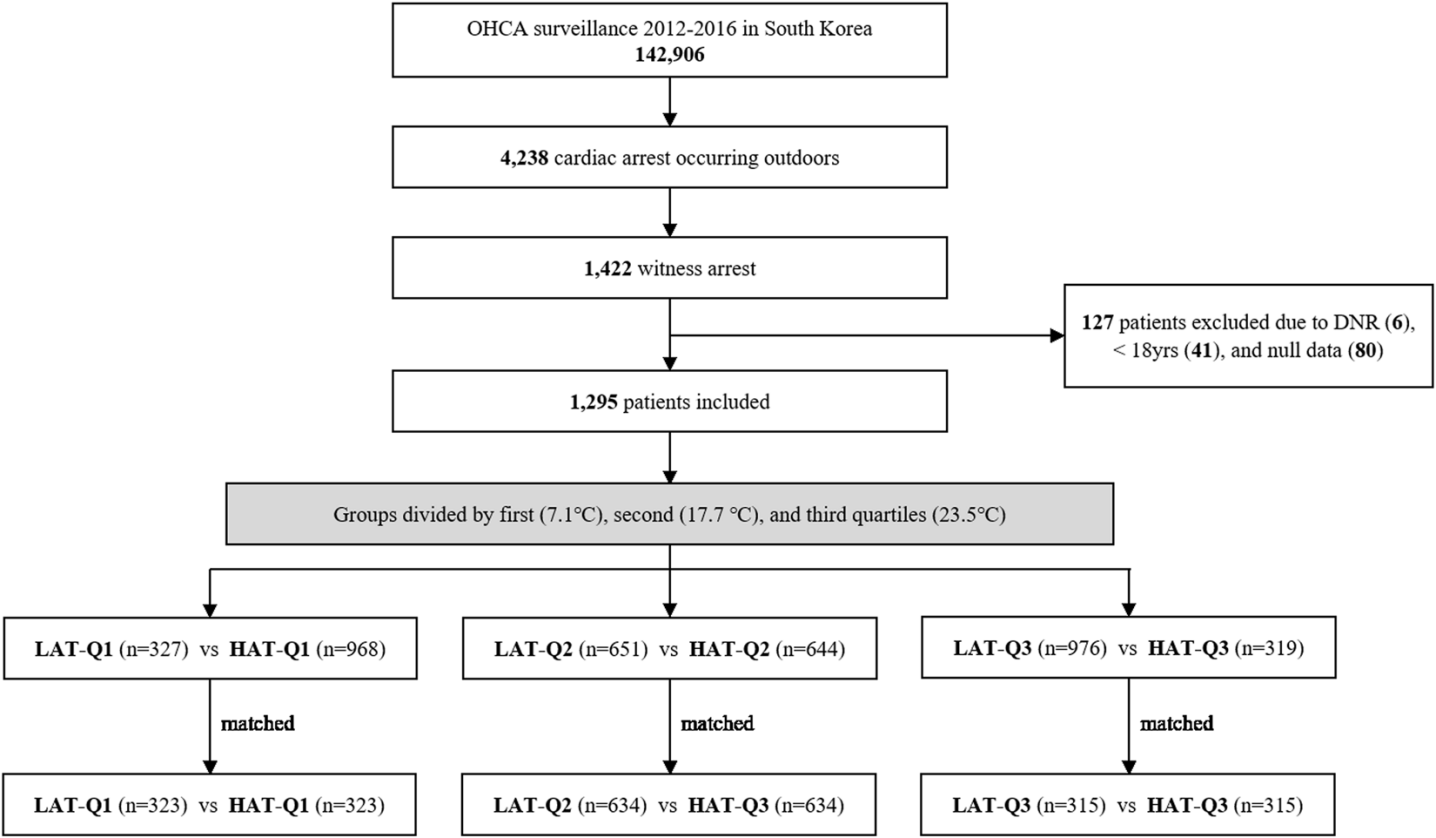
Flow diagram of study patients and study design. OHCA, out-of-hospital cardiac arrest; DNR, do not resuscitate; LAT, group of low ambient temperature; HAT, group of high ambient temperature; Q1, first quartile (7.1 °C); Q2, second quartile (17.7 °C); Q3, third quartile (23.5 °C).

We identified 142,906 patients between January 1, 2012, and December 31, 2016. Among them, 4,238 patients who had cardiac arrest occurring outdoors were enrolled in this study. After excluding unwitnessed cardiac arrest patients (n = 2,816), the patients (n = 6) with “do not resuscitate” orders, those (n = 41) younger than 18 years, and those (n = 80) with unavailable outcome data were additionally excluded. Finally, a total of 1,295 patients were included in this study (Fig. 1). The baseline characteristics of the patients are summarized in Table 1. The patients’ median age was 56 (47–65) years, and the percentages of bystanders performing CPR was 79.9%. Median time from cardiac arrest to emergency room (ER) arrival was 51 (35–78) minutes, and that from cardiac arrest to CPR discontinuation was 75 (59–97) minutes.

**Table 1:** Baseline characteristics.

Parameters	All (n = 1,295)	AT^a^				P value
		≤7.1 °C (n = 327)	>7.1 °C, ≤17.7 °C (n = 324)	>17.7 °C, ≤23.5 °C (n = 325)	>23.5 °C (n = 319)	
Age, year	56 (47–65)	59 (51–68)	56 (48–64)	58 (49–65)	53 (41–62)	**<0.001**
Gender, male	1,106 (85.4%)	271 (82.9%)	277 (85.5%)	282 (86.8%)	276 (86.5%)	0.163
Cause of arrest, cardiac	641 (49.5%)	187 (57.2%)	181 (55.9%)	160 (49.2%)	113 (35.4%)	**<0.001**
Bystander CPR	460 (79.9%)	86 (75.4%)	127 (80.9%)	119 (76.8%)	128 (85.3%)	0.108
Defibrillation	223 (70.8%)	60 (65.2%)	62 (75.6%)	53 (69.7%)	47 (73.4%)	0.382
Mechanical CPR	42 (3.2%)	10 (3.1%)	12 (3.7%)	12 (3.7%)	8 (2.5%)	0.712
ECPR	17 (1.3%)	3 (0.9%)	7 (2.2%)	2 (0.6%)	5 (1.6%)	0.887
PCI	31 (2.4%)	7 (2.1%)	8 (2.5%)	8 (2.5%)	8 (2.5%)	0.773
TTM	48 (3.7%)	12 (3.7%)	15 (4.6%)	9 (2.8%)	12 (3.8%)	0.735
Time (Arrest ~ ER arrival), min	51 (35–78)	50 (31–68)	51 (35–79)	50 (35–78.5)	54 (37–83.75)	0.204
Time (Arrest ~ stopping CPR), min	75 (59–97)	75 (59–94)	79 (60–97.75)	75 (58–100)	74 (58.5–97.75)	0.875
ROSC	261 (35%)	53 (27%)	65 (36.7%)	72 (37.3%)	71 (39.7%)	**0.012**
Survival to discharge	80 (6.2%)	17 (5.2%)	17 (5.2%)	29 (8.9%)	17 (5.3%)	0.488

Values are represented as median (interquartile range) or number (proportion).

^a^Four groups were categorized by each quartile value of AT (Q1 = 7.1 ^°^C; Q2 = 17.7 ^°^C; Q3 = 23.5 ^°^C).

*Abbreviations*: AT, ambient temperature; CPR, cardiopulmonary resuscitation; ECPR, extracorporeal membrane oxygenation cardiopulmonary resuscitation; PCI, percutaneous coronary intervention; TTM, target temperature management; ER, emergency room; ROSC, return of spontaneous resuscitation; Q1, first quartile; Q2, second quartile; Q3, third quartile.

### Comparisons of low and high AT groups by the standard of three quartiles

#### Low (LAT-Q1; below Q1) vs. high AT (HAT-Q1; above Q1) by the standard of 7.1 °C (Q1)

**Figure 2:**
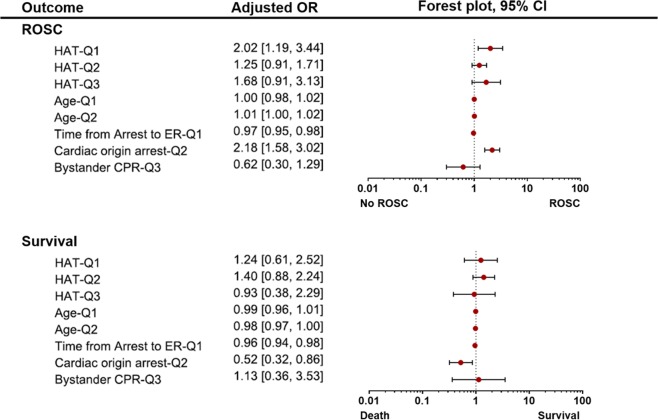
Forest plot for multivariable logistic regression analysis for outcomes. Each quartile standard of ambient temperature for the comparison of both low and high temperature groups is represented as Q1 (7.1 °C), Q2 (17.7 °C), Q3 (23.5 °C). To perform multivariate analysis, the logistic regression with ‘enter’ method was inderpently peformed for each ambient temperature quartile (Q1, Q2 and Q3) comparison. Age (Q1 and Q2), bystander CPR (Q3), cardiac cause of arrest (Q2), time from arrest to ER arrival (Q1) and HAT/LAT (Q1, Q2 and Q3) for each quartile standard were finally adjusted for each outcome (ROSC and survival to discharge). OR, odd ratios; CI, confidence interval; ROSC, return of spontaneous circulation; HAT-Q1, high ambient temperature group by the standard of first quartile temperature; Q1, first quartile; Q2, second quartile; Q3, third quartile; ER, emergency room; CPR, cardiopulmonary resuscitation.

In the comparison by the standard of 7.1 °C (Q1), there were significant differences in age and cardiac cause of arrest between unmatched LAT-Q1 (n = 327) and HAT-Q1 (n = 968) groups (Table 2). After 1:1 propensity score matching for these two covariates, the age and time from arrest to ER arrival remained unmatched between matched LAT-Q1 (n = 323) and HAT-Q1 (n = 323) groups (Table 3). In the univariate analysis for outcomes after matching, there were no significant differences between both matched groups in the ROSC and survival to discharge. However, in the multivariate analysis for matched groups, the ROSC was higher in the HAT-Q1 group than in the LAT-Q1 group (adjusted OR 2.02, 95% CI 1.19–3.44). There were no significant differences in the survival to discharge between both matched groups (Fig. 2).

**Table 2:** Comparison of unmatched groups divided by first quartile (7.1 °C), second quartile (17.7 °C), and third (23.5 °C) of ambient temperature.

Parameters	LAT-Q1 (n = 327)	HAT-Q1 (n = 968)	P value	LAT-Q2 (n = 651)	HAT-Q2 (n = 644)	P value	LAT-Q3 (n = 976)	HAT-Q3 (n = 319)	P value
Age, year	59 (51–68)	55 (46–64)	**<0.001**	57 (50–65)	55 (45–64)	**<0.001**	57 (49–65)	53 (41–62)	**<0.001**
Gender, male	271 (82.9%)	835 (86.3%)	0.134	541 (84.1%)	549 (86.6%)	0.214	817 (84.9%)	273 (86.7%)	0.449
Cause of arrest, cardiac	187 (57.2%)	454 (46.9%)	**0.001**	368 (57.2%)	273 (43.1%)	**<0.001**	528 (54.9%)	113 (35.9%)	**<0.001**
Bystander CPR	86 (75.4%)	374 (81.0%)	0.189	211 (78.7%)	245 (80.9%)	0.527	329 (78.0%)	127 (85.2%)	0.057
Defibrillation	60 (65.2%)	162 (73.0%)	0.169	122 (70.5%)	99 (71.7%)	0.814	174 (70.4%)	47 (73.4%)	0.638
Mechanical CPR	10 (3.1%)	32 (3.3%)	0.827	22 (3.4%)	19 (3.0%)	0.667	34 (3.5%)	7 (2.2%)	0.252
ECPR	3 (0.9%)	14 (1.4%)	0.584^a^	10 (1.6%)	7 (1.1%)	0.482	12 (1.2%)	5 (1.6%)	0.413^a^
PCI	7 (2.1%)	24 (2.5%)	0.967	15 (2.3%)	16 (2.5%)	0.825	23 (2.4%)	9 (2.5%)	0.882
TTM	12 (3.7%)	36 (3.7%)	0.729	27 (4.2%)	21 (3.3%)	0.405	36 (3.7%)	12 (3.8%)	0.957
Time (Arrest ~ ER arrival), min	50 (31–68)	50 (31–68)	0.080	50 (33–75)	51 (36–80.5)	0.304	50 (33–76)	54 (37–83.75)	0.102
Time (Arrest ~ stopping CPR), min	75 (59–94)	75 (59–94)	0.642	77 (59–95)	74 (58–98)	0.834	76 (59–96)	73.5(58.5–97.75)	0.591
ROSC	53 (27%)	208 (37.9%)	**0.006**	117 (31.6%)	141 (38.6%)	**0.047**	188 (33.6%)	70 (39.8%)	0.137
Survival to discharge	17 (5.2%)	63 (6.5%)	0.395	34 (5.3%)	45 (7.1%)	0.179	62 (6.4%)	17 (5.4%)	0.503

Values are represented as median (interquartile range) or number (proportion).

^a^Fisher’s exact test.

*Abbreviations*: LAT-Q1, group of low ambient temperature – groups divided by first quartile (7.1 °C); HAT-Q1, group of high ambient temperature – groups divided by first quartile (7.1 °C); LAT-Q2, group of low ambient temperature – groups divided by second quartile (17.7 °C); HAT-Q2, group of high ambient temperature – groups divided by second quartile (17.7 °C); LAT-Q1, group of low ambient temperature – groups divided by third quartile (23.5 °C); HAT-Q1, group of high ambient temperature – groups divided by third quartile (23.5 °C); CPR, cardiopulmonary resuscitation; ECPR, extracorporeal membrane oxygenation cardiopulmonary resuscitation; PCI, percutaneous coronary intervention; TTM, target temperature management; ER, emergency room; ROSC, return of spontaneous resuscitation.

**Table 3:** Comparison of matched groups divided by first quartile (7.1 °C), second quartile (17.7 °C), and third (23.5 °C) of ambient temperature.

Parameters	LAT-Q1 (n = 323)	HAT-Q1 (n = 323)	P value	LAT-Q2 (n = 634)	HAT-Q2 (n = 634)	P value	LAT-Q3 (n = 315)	HAT-Q3 (n = 315)	P value
Age, year	59 (51–68)	56 (47–66)	**0.010**	57 (49–65)	55 (45–64)	**0.001**	53 (42–62)	53 (41–62)	0.861
Gender, male	268 (83%)	278 (86.1%)	0.277	534 (84.2%)	549 (86.6%)	0.233	274 (87.0%)	273 (86.7%)	0.906
Cause of arrest, cardiac	187 (57.9%)	187 (57.9%)	1.000	359 (56.6%)	273 (43.1%)	**<0.001**	112 (35.6%)	113 (35.9%)	0.934
Bystander CPR	85 (75.9%)	139 (80.3%)	0.371	209 (78.6%)	245 (80.9%)	0.498	93 (72.7%)	127 (85.2%)	**0.010**
Defibrillation	60 (65.9%)	62 (75.6%)	0.163	119 (70.0%)	99 (71.7%)	0.739	40 (62.5%)	47 (73.4%)	0.185
Mechanical CPR	10 (3.1%)	10 (3.1%)	1.000	22 (3.5%)	19 (3.0%)	0.634	11 (3.5%)	7 (2.2%)	0.339
ECPR	3 (0.9%)	4 (1.2%)	1.000^a^	10 (1.6%)	7 (1.1%)	0.464	5 (1.6%)	5 (1.6%)	1.000
PCI	7 (2.2%)	9 (2.8%)	0.613	15 (2.4%)	16 (2.5%)	0.856	6 (1.9%)	8 (2.5%)	0.589
TTM	12 (3.7%)	10 (3.1%)	0.664	27 (4.3%)	21 (3.3%)	0.377	9 (2.9%)	12 (3.8%)	0.506
Time (Arrest ~ ER arrival), min	50 (31–68.75)	56 (37.75–80)	**0.046**	51 (33–75)	51 (36–80.5)	0.391	50 (35–78)	54 (37–83.75)	0.381
Time (Arrest ~ stopping CPR), min	75 (59–94)	75 (60.5–102)	0.248	77 (59–95)	74 (58–98)	0.808	78 (60–98)	73.5 (58.5–97.75)	0.210
ROSC	53 (27.2%)	63 (35.0%)	0.102	115 (31.5%)	141 (38.6%)	**0.044**	54 (30.0%)	70 (39.8%)	0.053
Survival to discharge	17 (5.3%)	23 (7.1%)	0.327	34 (5.4%)	45 (7.1%)	0.201	17 (5.4%)	17 (5.4%)	1.000

Values are represented as median (interquartile range) or number (proportion).

^a^Fisher’s exact test.

*Abbreviations*: LAT-Q1, group of low ambient temperature – groups divided by first quartile (7.1 °C); HAT-Q1, group of high ambient temperature – groups divided by first quartile (7.1 °C); LAT-Q2, group of low ambient temperature – groups divided by second quartile (17.7 °C); HAT-Q2, group of high ambient temperature – groups divided by second quartile (17.7 °C); LAT-Q1, group of low ambient temperature – groups divided by third quartile (23.5 °C); HAT-Q1, group of high ambient temperature – groups divided by third quartile (23.5 °C); CPR, cardiopulmonary resuscitation; ECPR, extracorporeal membrane oxygenation cardiopulmonary resuscitation; PCI, percutaneous coronary intervention; TTM, target temperature management; ER, emergency room; ROSC, return of spontaneous resuscitation.

#### LAT-Q2 vs. HAT-Q2 by the standard of 17.7 °C (Q2)

In the comparison by the standard of 17.7 °C (Q2), there were significant differences in age and cardiac cause of arrest between unmatched LAT-Q2 (n = 651) and HAT-Q2 (n = 644) groups (Table 2). After 1:1 propensity score matching for these two covariates, the age and cardiac cause of arrest remained unmatched between matched LAT-Q2 (n = 634) and HAT-Q2 (n = 634) groups (Table 3). In the univariate analysis for outcomes after matching, the ROSC was higher in the HAT-Q2 group than in the LAT-Q2 group (38.6% vs. 31.5%, p = 0.044). There were no significant differences between both matched groups in survival to discharge. However, in the multivariate analysis for matched groups, no significant differences in ROSC and survival to discharge were found between the HAT-Q2 and LAT-Q2 groups (Fig. 2).

### LAT-Q3 vs. HAT-Q3 by the standard of 23.5 °C (Q3)

In the comparison by the standard of 23.5 °C (Q3), there were significant differences in age and cardiac cause of arrest between unmatched LAT-Q3 (n = 976) and HAT-Q3 (n = 319) groups (Table 2). After 1:1 propensity score matching for these two covariates, the bystander CPR remained unmatched between matched LAT-Q3 (n = 315) and HAT-Q3 (n = 315) groups (Table 3). In the univariate analysis for outcomes after matching, there were no significant differences between both matched group in the ROSC and survival to discharge. Nevertheless, in the multivariate analysis for matched groups, the HAT-Q3 group showed no significant benefit for ROSC and survival to discharge compared with the LAT-Q3 group (Fig. 2).

In addition, further analysis of four mutually exclusive AT group was performed. ROSC is significantly lower in the AT group of ≤7.1 °C than in the AT group of >23.5 °C. Survival to discharge is significantly higher in the AT group of ≤7.1 °C and 17.7 °C < AT ≤ 23.5 °C than in the AT group of >23.5 °C (Supplementary Tables 1 and 2).

## Discussion

This nationwide and large population-based study investigated the association of AT with the outcomes in patients with witnessed OHCA occurring outdoor. No obvious correlation existed between the AT and patient outcomes such as sustained ROSC or survival to discharge. We only identified that the AT group of <7.1 °C had lower ROSC than the AT group ≥ 7.1 °C. This result might have originated from the significantly lower ROSC in the AT group of <7.1 °C than in the AT group of ≥23.5 °C.

The most common cause of OHCA is cardiovascular disease^12,13^. Physiological control of the human body can increase cardiac work and sympathetic activation^14^. During winter with cold weather, markers of the sympathetic nervous system, such as epinephrine and norepinephrine, increase and are associated with heart rate variability^15–17^. Activation of the coagulation system and the sympathetic nervous system contributes to the occurrence of arrhythmias^18^. Moreover, cold conditions could cause vasoconstriction, which will increase the heart workload. These actions may reduce the ischemic threshold and cause sudden collapse^18^.

Previous studies have shown an interest in the relationship between the seasonal AT and cardiovascular mortality^8–11^. Some studies have shown a negative correlation between seasonal AT and cardiovascular mortality^19,20^, whereas others have shown that seasonal variability of outside temperature has an influence on cardiac arrest^8–10,21^. Fukuda *et al*. showed that the increase of the AT might affect the neurologic outcome in OHCA patients^8^. They studied all types of cardiac arrest without taking into consideration the location of arrest (indoor and outdoor) and monthly temperature^8^. Moreover, they found that lower AT induced more hypothermic state, and cardiac arrest at the hypothermic state was a good neurological opportunity after ROSC because of its protective effect on the vital organs^8^, but the study did not show such a result. Additionally, they showed that the cold season was associated with significantly poorer neurologic outcomes^8^. However, it was uncertain to what degree of AT has affected the change in body temperature.

We studied the association of AT with the prognosis of OHCA occurring outdoor, but we could not detect the body temperature of the patients when they collapsed. AT can affect the skin temperature of arrest patients, but given that there is no linear correlation between the skin and core temperatures due to the thermoregulation of the body system, it is necessary to consider the effect of core temperature to predict the outcome of OHCA patients^22^.

Skin temperature increases or decreases depending on the fluctuation of AT^22^. However, core temperature remains fairly constant through thermoregulation^22^. In normal physical conditions, AT for the body affects a series of processes. A high AT suppresses the sympathetic centres of the hypothalamus, and heat is evaporated through sweating, which causes marked dilation of skin vessels in almost all parts of the body^22,23^. On the other hand, when the AT is low, sympathetic stimulation causes contraction of the skin vessels with an insulating effect that protects the deep organs from heat loss^22–24^. This effect varies depending on the age of the individual. OHCA usually occurs during poor weather conditions^10^, and its occurrence is related to hot^25–27^ or cold weather^28,29^; mortality increases significantly in the elderly. A previous study has demonstrated that aging increases susceptibility to temperature extremes, which is related to the thermoregulatory capacity in the elderly^30^. After statistically adjusting the age effect in this study, cardiac origin arrest showed significant results in terms of outcome. The high odds of cardiac origin arrest in ROSC was considered to be due to whether defibrillation was performed (cardiac origin 28.0%, non-cardiac origin 6.5%). In addition, survival might be considered as a result of in-hospital treatment, such as cardiac intervention or therapeutic hypothermia.

This study has some limitations. First, even though matched analyses were performed, there were no significant differences in the survival and ROSC outcomes among the temperature categories in most analyses. It suggests that the application of propensity score matching may be ineffective and underpowered in the interpretation for this study. Second, it was not easy to reflect the individuals’ disease information and characteristics. Given that the investigators obtain the information through a chart review after the cardiac arrest has occurred, detailed disease information is likely to be missed. Moreover, we did not obtain information related to occupation and outdoor activity during the hot and cold climates. Third, it is difficult to obtain information about the procedures and treatments in the hospital. Additionally, the source data did not provide any information about whether the reversible cause of arrest was well resolved. Fourth, the following outdoor or indoor circumstances of patients could affect patient outcomes: the duration of the patients staying outdoor; indoor temperature via heat or air conditioner before they went outdoor; and sudden changes of AT. However, these factors are not included in the analysis owing to the lack of available data for this study, which could be critical. Fifth, the use of mean daily temperature for each city or province can mask the effect of temperature change during the day (morning vs. evening vs. night). It is possible that a patient had their arrest in the morning when the temperature was colder. The actual temperature may differ within cities or provinces. Finally, the nationwide data of this study was only confined to South Korea. If the patients were from other countries, with different races, or from the different medical system, the results might be different. In terms of AT, South Korea is located in the mid-latitude temperate climatic zone. Accordingly, other countries located in the equator or polar region may have different results.

In conclusion, this nationwide, population-based study demonstrates that no obvious correlation existed between the AT and patient outcomes such as sustained ROSC or survival to discharge. This study only suggested the possibility of the difference of AT impacting ROSC and survival. To clarify the association of AT with the prognosis of OHCA patients in further study, the investigators need to consider more delicate information affecting body temperature.

## Methods

### Study design and participants

Out-of-Hospital Cardiac Arrest Surveillance (OHCAS) is a nationwide, population-based database from the Korea Centres for Disease Control and Prevention in Korea. Since 2012, all acute cardiac arrest patients transferred to medical institutions via EMS were included. Approximately 30,000 patients are included per year. An investigator from the institution visited the medical institution to review the patients’ medical records and confirmed several items according to the Utstein Style and Resuscitation Outcome Consortium (ROC) Project.

This study population included adult patients aged 18 years and older with witnessed OHCA occurring outdoors that could have been affected by the AT, between January 2012 and December 2016. Exclusion criteria were as follows: indoor arrest, arrest in an unknown place, unwitnessed arrest, patients with “do not resuscitate” orders, and patients aged younger than 18 years.

### Meteorological data

Meteorological data were retrieved from the database of the Korea Meteorological Administration. The data included the mean temperature of 17 metropolitan cities and provinces divided by governmental administrative adjustment in the Republic of Korea. The size of the metropolitan area is 464 km^2^ to 1,063 km^2^, and the size of the province is 1,850 km^2^ to 19,032 km^2^ ^31^. The Republic of Korea is located geographically in the mid-latitude temperate climatic zone, and the four seasons of spring, summer, fall, and winter are conspicuous. The average annual temperature is 10 °C–15 °C, with the warmest temperature of 23 °C–26 °C and the coldest temperature of −6 °C–3 °C^32^. In the present study, the mean daily temperature of each city or province was matched with each cardiac arrest patient data, considering the day and place of cardiac arrest.

### Outcome measures

The primary outcome measure was the sustained ROSC for at least 20 minutes in the hospital. Secondary outcomes included survival to hospital discharge.

### The groups of LAT and HAT, divided by the first, second, and third quartiles

Patients who met the inclusion criteria among all OHCA patients were finally included in the analysis and categorized into the following groups according to the AT in which OHCA occurred. The groups were compared separately according to the AT in three quartiles: low (LAT-Q1; below Q1) vs. high AT (HAT-Q1; above Q1) (divided by the first quartile (Q1) = 7.1 °C), LAT-Q2 vs. HAT-Q2 (divided by the second quartile (Q2) = 17.7 °C), and LAT-Q3 vs. HAT-Q3 (divided by the third quartile (Q3) = 23.5 °C) (Fig. 1). We attempted to obtain the cut-off of AT related to favourable outcomes by receiver operating characteristic (ROC) curve. In the analysis by ROC curve, we failed to obtain the optimal value or extract it from previous studies (Supplementary Fig. 1).

### Statistics

Data analyses were performed using the Statistical Package for the Social Sciences (SPSS), version 21.0 KO for Windows (SPSS Inc., Chicago, IL, USA) and R version 3.3.2 (http://www.web-r.org) software. Descriptive statistics were used to describe the baseline characteristics of the study participants and to present categorical variables as frequencies and percentages. Non-normally distributed data were presented as medians with interquartile ranges (IQR). In the univariate analysis, the Mann-Whitney U test was used for comparison of continuous variables and Chi-squared or Fisher’s exact test for categorical variables.

Propensity score matching analysis was performed to overcome the bias arising from the lack of randomisation as a consequence of the different co-variable distributions among patients who were included in the LAT and HAT groups. The predicted values were then used to obtain 1:1 nearest-neighbour matching. Patients for whom the propensity score matching analysis could not be matched were excluded from the outcome comparisons of the matching groups. To identify predictors for outcomes (ROSC and survival to discharge), statistically significant covariates between both matched groups were only included and evaluated by multivariate analysis. To perform multivariate analysis, the logistic regression with ‘enter’ method was indepedently performed for each AT quartile (Q1, Q2 and Q3) comparison. Age (Q1 and Q2), bystander CPR (Q3), cardiac cause of arrest (Q2), time from arrest to ER arrival (Q1) and HAT/LAT (Q1, Q2 and Q3) for each quartile standard were finally adjusted. The results were represented as adjusted odds ratios (95% CI) and merged into a forest plot. A p-value of <0.05 was considered statistically significant.

### Ethics statement

The protocol of the study was approved by the institutional review board of Kangnam Sacred Heart Hospital (IRB No. HKS 2018-07-023), and participants gave written informed consent. All participants’ records were anonymized before being accessed by the authors, and all methods were carried out in accordance with the approved guidelines and regulations.

## Supplementary information

Supplementary Figure

Supplementary Tables

## Data Availability

The datasets generated and analysed during the current study are available from the corresponding author on reasonable request.
