# Supplementary material for: Association of ambient temperature with the outcomes in witnessed out-of-hospital cardiac arrest patients: a population-based observational study
Source: Sci Rep. 2019 Sep 16;9:13417. doi: 10.1038/s41598-019-50074-7 (PMC6746864; doi:10.1038/s41598-019-50074-7)

**Association of ambient temperature with the outcomes in witnessed out-of-hospital cardiac arrest patients: a population-based observational study**

Chiwon Ahn^1,2,†^, Jihoon Kim^3,†^, Wonhee Kim^2,4,^*, In Young Kim^2^, Hyun Young Choi^4^, Jae Guk Kim^4^, Bongyoung Kim^5^, Shinje Moon^6^, Hyungoo Shin^7^, Juncheol Lee^8^

^1^Department of Emergency Medicine, Armed Forces Yangju Hospital, Yangju, Republic of Korea
^2^Department of Biomedical Engineering, College of Medicine, Hanyang University, Seoul, Republic of Korea
^3^Department of Thoracic and Cardiovascular Surgery, College of Medicine, Hallym University, Chuncheon, Republic of Korea
^4^Department of Emergency Medicine, College of Medicine, Hallym University, Chuncheon, Republic of Korea
^5^Department of Internal Medicine, College of Medicine, Hanyang University, Seoul, Republic of Korea
^6^Department of Internal Medicine, College of Medicine, Hallym University, Chuncheon, Republic of Korea
^7^Department of Emergency Medicine, Hanyang University Guri Hospital, Guri, Republic of Korea
^8^Department of Emergency Medicine, Armed Forces Capital Hospital, Seongnam, Republic of Korea
 ^†^Ahn and Kim contributed equally to this work.

***Corresponding author:** Wonhee Kim, MD., PhD.

Department of Emergency Medicine, College of Medicine, Hallym University, Chuncheon, Republic of Korea

1, Singil-ro, Yeongdeungpo-gu, Seoul, 07441, Republic of Korea

Tel: +82-2-829-5561

Fax: +82-2-842-4217

E-mail: wonsee02@gmail.com

**Supplementary Figure 1.** Receiver Operating Characteristic (ROC) curve of ambient temperature in ROSC, Survival and Good Neurologic outcome.


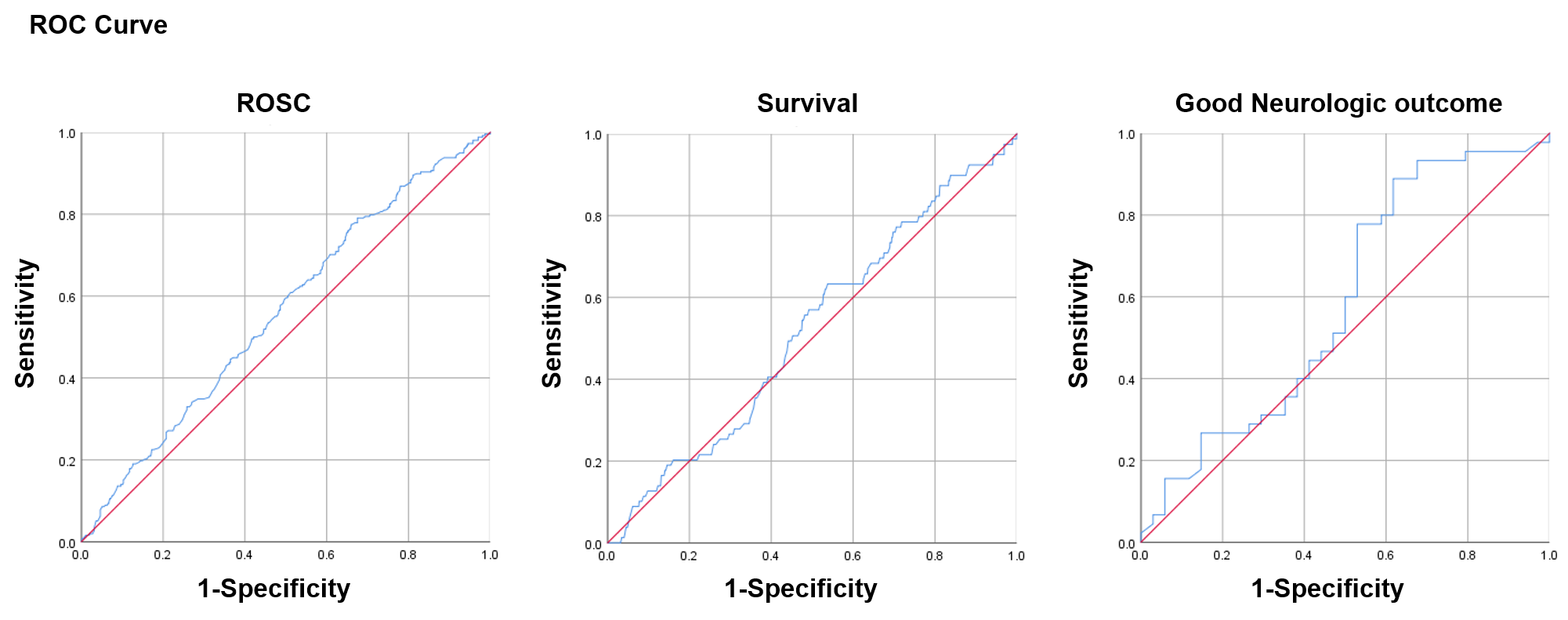

Supplement: Supplementary file 1 — Supplementary Figure [file 41598_2019_50074_MOESM1_ESM.docx]
