# Supplementary material for: Association of ambient temperature with the outcomes in witnessed out-of-hospital cardiac arrest patients: a population-based observational study
Source: Sci Rep. 2019 Sep 16;9:13417. doi: 10.1038/s41598-019-50074-7 (PMC6746864; doi:10.1038/s41598-019-50074-7)
Supplement: Supplementary file 2 — Supplementary Tables [file 41598_2019_50074_MOESM2_ESM.docx]

**Association of ambient temperature with the outcomes in witnessed out-of-hospital cardiac arrest patients: a population-based observational study**

Chiwon Ahn^1,2,†^, Jihoon Kim^3,†^, Wonhee Kim^2,4,^*, In Young Kim^2^, Hyun Young Choi^4^, Jae Guk Kim^4^, Bongyoung Kim^5^, Shinje Moon^6^, Hyungoo Shin^7^, Juncheol Lee^8^

^1^Department of Emergency Medicine, Armed Forces Yangju Hospital, Yangju, Republic of Korea
^2^Department of Biomedical Engineering, College of Medicine, Hanyang University, Seoul, Republic of Korea
^3^Department of Thoracic and Cardiovascular Surgery, College of Medicine, Hallym University, Chuncheon, Republic of Korea
^4^Department of Emergency Medicine, College of Medicine, Hallym University, Chuncheon, Republic of Korea
^5^Department of Internal Medicine, College of Medicine, Hanyang University, Seoul, Republic of Korea
^6^Department of Internal Medicine, College of Medicine, Hallym University, Chuncheon, Republic of Korea
^7^Department of Emergency Medicine, Hanyang University Guri Hospital, Guri, Republic of Korea
^8^Department of Emergency Medicine, Armed Forces Capital Hospital, Seongnam, Republic of Korea
 ^†^Ahn and Kim contributed equally to this work.

***Corresponding author:** Wonhee Kim, MD., PhD.

Department of Emergency Medicine, College of Medicine, Hallym University, Chuncheon, Republic of Korea

1, Singil-ro, Yeongdeungpo-gu, Seoul, 07441, Republic of Korea

Tel: +82-2-829-5561

Fax: +82-2-842-4217

E-mail: wonsee02@gmail.com

**Supplementary Table 1.** Multivariate logistic regression for ROSC

| Factors | OR (95% CI) | | | |
| --- | --- | --- | --- | --- |
| Gender, male | 0.64 (0.39-1.05) | | | |
| Age, year | 1.02 (1.00-1.03) | | | |
| Cause of arrest, cardiac | **3.31 (2.17-5.10)** | | | |
| Time (Arrest ~ ER), min | **0.98 (0.98-0.99)** | | | |
| Ambient temperature  ≤7.1ºC  >7.1ºC, ≤17.7ºC  >17.7ºC, ≤23.5ºC  >23.5ºC | Reference  1.66 (0.96-2.91)  1.55 (0.89-2.73)  **2.10 (1.18-3.78)** | 0.60 (0.34-1.05)  Reference  0.93 (0.54-1.60)  1.27 (0.73-2.20) | 0.65 (0.37-1.13)  1.07 (0.63-1.84)  Reference  1.36 (0.79-2.35) | **0.48 (0.26-0.85)**  0.79 (0.45-1.37)  0.74 (0.43-1.27)  Reference |

Abbreviations: ROSC, return of spontaneous circulation; OR, odds ratio; CI, confidence interval; ER, emergency room

**Supplementary Table 2**. Multivariate logistic regression for survival

| Factors | OR (95% CI) | | | |
| --- | --- | --- | --- | --- |
| Age, year | 0.98 (0.95-1.00) | | | |
| Time (Arrest ~ ER), min | **0.95 (0.92-0.97)** | | | |
| Ambient temperature  ≤7.1ºC  >7.1ºC, ≤17.7ºC  >17.7ºC, ≤23.5ºC  >23.5ºC | Reference  0.45 (0.14-1.29)  0.85 (0.33-2.20)  **0.22 (0.05-0.77)** | 2.22 (0.78-6.90)  Reference  1.90 (0.66-5.87)  0.49 (0.12-1.82) | 1.17 (0.45-3.06)  0.53 (0.17-1.52)  Reference  **0.26 (0.06-0.87)** | **4.52 (1.30-18.89)**  2.04 (0.55-8.52)  **3.86 (1.14-15.56)**  Reference |

Abbreviations: OR, odds ratio; CI, confidence interval; ER, emergency room
